# Phenotypes of Dravet Syndrome

**DOI:** 10.15844/pedneurbriefs-30-5-1

**Published:** 2016-05

**Authors:** Rebecca Garcia-Sosa, Linda C. Laux

**Affiliations:** 1 Division of Neurology, Ann & Robert H. Lurie Children’s Hospital of Chicago, Chicago, IL; 2 Departments of Pediatrics and Neurology, Northwestern University Feinberg School of Medicine, Chicago, IL

**Keywords:** Dravet syndrome, Sodium channels, Epilepsy, Behavior

## Abstract

Researchers from the University of Washington in Seattle studied selective heterozygous and homozygous deletions of the voltage gated sodium channel (Nav1.1) in parvalbumin (PV) or somato-statin (SST) expressing interneurons.

Researchers from the University of Washington in Seattle studied selective heterozygous and homozygous deletions of the voltage gated sodium channel (Nav1.1) in parvalbumin (PV) or somato-statin (SST) expressing interneurons. These GABAergic inhibitory interneurons account for a large number of total interneurons and are known to play an important role in the pathophysiology of Dravet syndrome (DS). To study the contributions of the interneuron subtypes to the different physiological phenotypes, they obtained electrophysiological recordings from mice with selective mutations. Haploinsufficiency of Nav1.1 in either class of interneuron (PV-expressing or SST-expressing) resulted in reduced excitability, which was observed in both cortical and hippocampal interneurons. Selective deletion of Nav1.1 on PV or SST-expressing interneurons independently was sufficient to cause thermally induced seizures and mild spontaneous seizures. When the same deletion is present in both interneurons, there is synergistic seizure susceptibility with earlier onset and longer duration seizures. Homozygous deletion of Nav1.1 in PV-expressing interneurons causes a more severe epileptic phenotype when compared to the same deletion in SST interneurons. Furthermore, complete deletion of Scn1a in both interneurons appears to have synergistic effects and result in a severe epileptic phenotype with more frequent premature death.

Additionally, investigators studied the varying behavioral phenotypes and found that deletion of Nav1.1 in SST-expressing interneurons contributes to hyperactivity while deletion in PV-expressing interneurons results in impaired social interactions. Together, deletion of Nav1.1 in both interneurons causes impaired long term fear memory. [1]

COMMENTARY. The incidence of SCN1A mutation associated DS in the United States was recently reported as high as 1 per 20,900 births, much higher than previously thought [2] and thus mandates increased awareness and understanding of the disease. SCN1A mutations produce a clinical spectrum of epilepsy varying from generalized epilepsy with febrile seizures plus (GEFS +) to the most severe end of the spectrum, DS or severe myoclonic epilepsy of infancy (SMEI) [3]. Almost 700 mutations have been identified in the SCN1A gene associated with DS alone, as well as potential genetic modifiers such as SCN9A variants [4].

Studies like this one attempt to dissect the genetics behind the neuropsychological heterogeneity and could lead to better counseling regarding prognosis at the time of diagnosis as well as development of more targeted therapies. It would also further clarify the epileptic encephalopathy vs channelopathy controversy that has been proposed to contribute to the varying profiles in DS [5].
